# Static Hand Gesture Recognition Using Capacitive Sensing and Machine Learning

**DOI:** 10.3390/s23073419

**Published:** 2023-03-24

**Authors:** Frazer Noble, Muqing Xu, Fakhrul Alam

**Affiliations:** Department of Mechanical and Electrical Engineering, School of Food and Advanced Technology, College of Sciences, Auckland Campus, Auckland 0632, New Zealand; f.k.noble@massey.ac.nz (F.N.); muqing.xu@outlook.co.nz (M.X.)

**Keywords:** hand gesture recognition, Human-to-Machine Interface, capacitive sensing, machine learning

## Abstract

Automated hand gesture recognition is a key enabler of Human-to-Machine Interfaces (HMIs) and smart living. This paper reports the development and testing of a static hand gesture recognition system using capacitive sensing. Our system consists of a 6×18 array of capacitive sensors that captured five gestures—Palm, Fist, Middle, OK, and Index—of five participants to create a dataset of gesture images. The dataset was used to train Decision Tree, Naïve Bayes, Multi-Layer Perceptron (MLP) neural network, and Convolutional Neural Network (CNN) classifiers. Each classifier was trained five times; each time, the classifier was trained using four different participants’ gestures and tested with one different participant’s gestures. The MLP classifier performed the best, achieving an average accuracy of 96.87% and an average $F_{1}$ score of 92.16%. This demonstrates that the proposed system can accurately recognize hand gestures and that capacitive sensing is a viable method for implementing a non-contact, static hand gesture recognition system.

## 1. Introduction

Hand gestures are an important aspect of human interaction and are used by people to communicate using non-verbal, visual cues. Automated hand gesture recognition systems have a multitude of applications in gaming, virtual and augmented reality, home automation, and Human-to-Machine Interfaces (HMIs) [1]. Hand gesture recognition systems can provide contactless interaction with machines and equipment. One of the lessons learned during the pandemic is that machines that are used by many, e.g., elevators, vending machines, etc., can spread the SARS-CoV-2 virus and would benefit from contactless operation [2].

Wearable device-based gesture recognition systems have shown promising results [3]. However, they are not contactless and can be inconvenient [4], resulting in a negative user experience due to the requirement of having to wear a sensor system, e.g., a glove [5] or band [6]. Vision-based gesture recognition systems have been heavily investigated, e.g., see the review article [7]. These techniques utilize a camera-based system for image and/or video acquisition. While such systems are very accurate within controlled environments and provide contactless recognition, their performance can be adversely impacted by occlusion, busy backgrounds, poor illumination, etc. In addition, camera-based systems can also be perceived to be intrusive to privacy, especially in homes [8], and people with disability, one of the intended beneficiary groups, feel that the presence of a camera brings unwelcome attention to their disability [9].

Alternative techniques for hand gesture recognition have therefore started to gain traction. Table 1 summarizes the salient points of such recent works. Researchers have investigated various sensing modalities, e.g., visible light sensing using Photodiodes (PDs) [4,10] and solar cells [11,12], Wi-Fi signal sensing [13,14], micro-Doppler signatures acquired from low-cost Doppler radars [2,15,16], etc., for hand gesture recognition. One of the inherent limitations of these techniques is that they are only capable of recognizing dynamic hand gestures [17], where the hand is making a sequence of movements. However, for many applications, it is only necessary to recognize static gestures with the hand shape or posture remaining stationary [18]. Since only a single “image” is fed as the input to the classifier, the static approach has the advantage of having a lower computational cost [19].

Capacitive sensing [20], sometimes termed electric field sensing, techniques underpin many human sensing applications ranging from localization [21], subject recognition [22], and occupancy estimation [23]. It has also shown the ability to provide both dynamic [9] and static [18] gesture recognition in a contactless manner. In this paper, we present a sensing system that uses a capacitive sensor array to capture static hand gestures in a contactless manner.

A trained classifier is required to identify the various hand gestures from the captured data. The literature shows that a variety of Machine Learning (ML)-based classifiers have been employed, with neural-network-based approaches being the most popular [1]. In this paper, we employ Multi-Layer Perceptron (MLP) and Convolutional Neural Network (CNN) neural-network-based multi-class classifiers to identify hand gestures. The performance of these neural-network-based classifiers was benchmarked against Naïve Bayes and Decision Tree classifiers. Given that the hand gestures were static with no temporal features, we did not consider Recurrent Neural Network (RNN)-based classification for our work.

Our work on the proposed hand gesture recognition system using capacitive sensing offers the following novel contributions:

- It is one of the first reported works that achieve contactless, static hand gesture recognition without using a camera. We developed a novel, bespoke system using a capacitive sensor array and a neural-network-based classifier.
- The performance, unlike many of the reported works in the literature, was tested with “unseen” subjects and achieved high accuracy.

The remainder of this article is organized as follows: Materials and Methods, where we describe our system’s components, the data collected, and our experimental process; Results, where we present the results from the experiments; and Conclusion and Future Works, where we discuss the presented results and propose future work.

## 2. Materials and Methods

In this section, the proposed gesture recognition system, data collected, classifiers used, training process, and metrics used are presented and described.

### 2.1. System Overview

**Figure 1:**
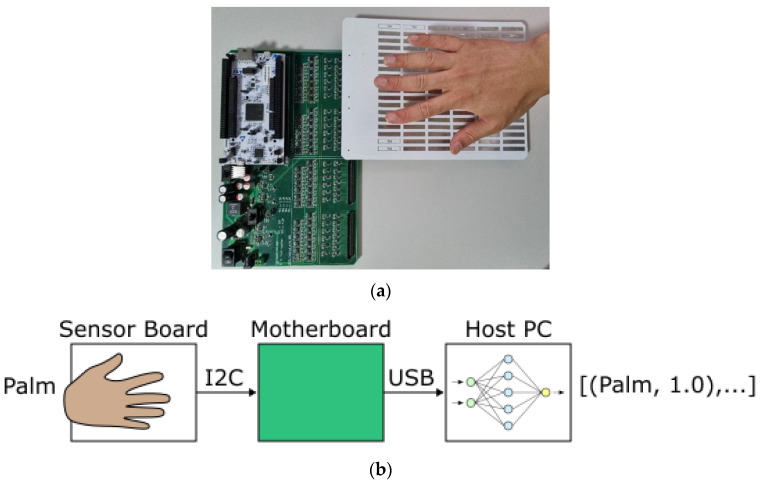
The proposed system: (**a**) Motherboard and connected Sensor Board; (**b**) Gesture recognition pipeline.

The proposed system consists of three parts: (1) the Sensor Board, (2) the Motherboard, and (3) the Classifier. The Sensor Board consists of an array of sensing pads and male board connectors. The Motherboard consists of power electronics, a Microcontroller (MCU), capacitance-to-digital converters, and female board connectors. The classifier is a program running on the host PC. The Sensor Board connects with the Motherboard using the board connectors (see Figure 1a). The Sensor Board’s sensing pads and the Motherboard’s capacitance-to-digital converters form the basis of the system’s capacitive sensors. Each sensor’s output is sent to the MCU via I2C. The MCU aggregates all the data into a single data frame, which is sent to the host PC via a USB-to-serial communication (COM) port. The Classifier processes the data and predicts what gesture is being made (see Figure 1b).

### 2.2. Sensor Board

**Figure 2:**
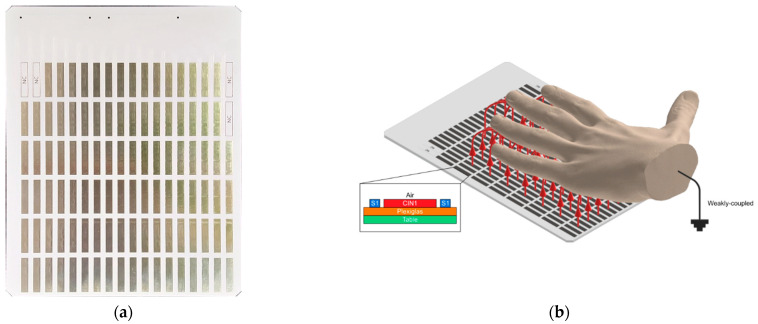
Sensor Board: (**a**) The Sensor Board; (**b**) Illustration of the loading mode capacitance.

The Sensor Board is a two-layer PCB that uses capacitance to detect the presence of a hand. It consists of a 6×18 array of copper sensing pads (see Figure 2a).

Its design is based on the formation of loading mode capacitance. The concept is illustrated in Figure 2b. When a hand is place over the board, it, and each sensing pad, form two plates of a capacitor, which can be expressed as:

(1) $$C=\varepsilon \frac{A}{d^{\gamma }}\;,$$

where C is the capacitance, A is the overlapped area of the two “plates”, ε is the permittivity of air, d is the distance between the plates, and γ is an environmental parameter [24].

When capacitance between the hand and the Sensor Board is measured, it results in an array of capacitance values that can be interpreted as a low-resolution “capacitive image”.

The size of the sensing pads affects the detectable range of the sensors and the spatial resolution of the image. A trade-off must be made between pad size, sensitivity, noise immunity, and coupling effects [25]. After considering these factors, pads of 5 mm×27 mm were chosen for the final design.

### 2.3. Motherboard

**Figure 3:**
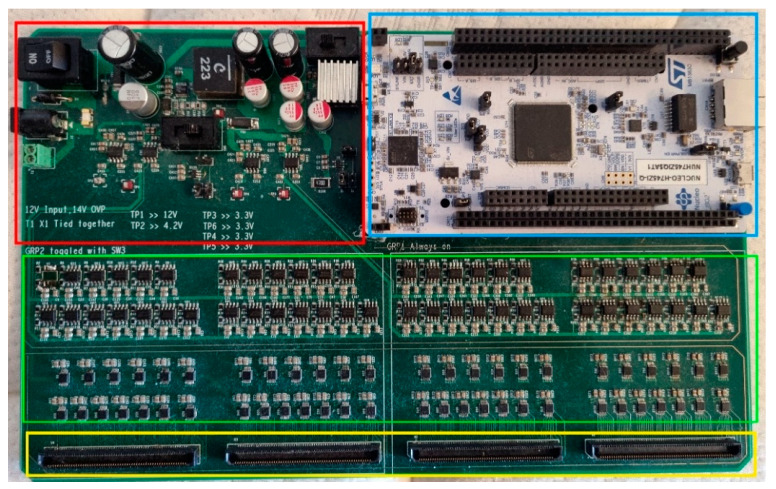
Motherboard. The Power Supply section (red) provides power for the whole board. The MCU section (blue) contains an STM32F7 Nucleo development board, which is used for data processing and communication with the host PC. The Sensor section (green) contains FDC1004 capacitance-to-digital converters and STM32G03 MCUs, which are used to collect capacitance data from the Sensor Board and transfer it to the STM32F7. The female board connectors (yellow) are used to connect the Sensor Board to the Motherboard.

The Motherboard (please see Figure 3) is a four-layer PCB that connects to the Sensor Board and communicates with the host PC. It consists of the Power Supply section, the Microcontroller (MCU) section, the Sensor section, and the female board connectors. EdgeRate connectors from Samtec [26] allow the Sensor Board to connect to the Motherboard. The Motherboard reads the capacitance values from the Sensor Board, processes the data, and communicates with the host PC via the MCU’s USB-to-serial COM port.

#### 2.3.1. Power Supply

**Figure 4:**
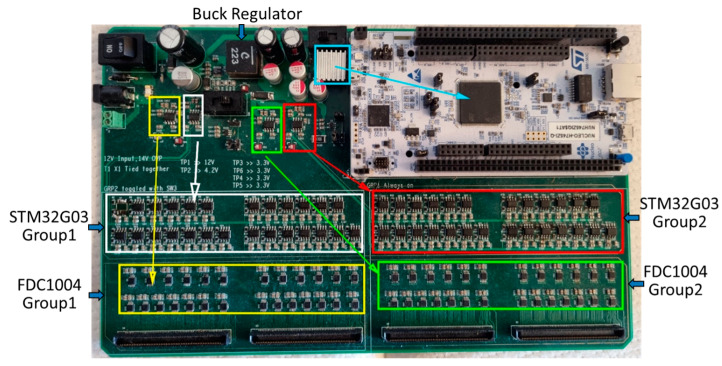
The LDO regulators powering the Motherboard’s sections. The 5 V LDO (teal) powers the MCU section; 3.3 V LDO 1 and 2 (yellow and white) power the Sensor section’s FDC1004 and STM32G03 Group 1s; 3.3 V LDO 3 and 4 (green and red) power the Sensor section’s FDC1004 and STM32G03 Group 2s.

The Power Supply section is made up of one 5 V Low Dropout (LDO) regulator, one 4.15 V buck converter, and four 3.3 V LDO regulators. It was designed based on noise and voltage regulation requirements and extensive simulations were performed to ensure the stability of the system. The 12 V input to the Motherboard is connected to the 5 V LDO regulator and the buck converter. A heat sink is used with the 5 V LDO regulator to dissipate heat. The buck converter is connected to the four 3.3 V LDO regulators. This two-step conversion (12 V to 4.15 V to 3.3 V) was deemed to be a good compromise between the safety margin of the 3.3 V LDO regulators’ operation limits and the heat generated. In addition, the buck regulator is protected by a fuse and a Zener diode, and a ferrite bead is used to mitigate the buck converter’s output noise. The 5 V LDO is used to power the MCU section. The 3.3 V LDOs are used to power the Sensor section’s STM32G03 MCUs and FDC1004 converters. Each of the 3.3 V LDO regulators power half of the STM 32G03s or half of the FDC1004s. This split was used to isolate high-frequency signals generated by the STM32G03s internal PLL and buck converters. Figure 4 illustrates the LDO regulators and the Motherboard’s sections they power.

#### 2.3.2. MCU Section

The MCU section is made up of an STM32F7 Nucleo development board. The STM32F7 combines the data from all the STM32G03 MCUs and sends it to the host PC. The communication between the STM32G03s and the STM32F7 is accomplished using a custom-designed bit-bang transmission scheme.

#### 2.3.3. Sensor Section

The Sensor section of the system is made up of 52 FDC1004 capacitance-to-digital converters from Texas Instruments. Each FDC1004 has four sensors, allowing the system to have up to 208 individual capacitive sensors. The FDC1004s communicate with the system using the I2C protocol. To manage this communication, the system uses one STM32G03 MCU per FDC1004.

The FDC1004s are used to measure capacitance by generating an excitation signal that is sent to the sensing pads. This signal can cause noise for nearby sensors, so a “sweep” is performed where each FDC1004 is turned on one at a time to collect data from its four sensor pads, then turned off. This process is repeated for each FDC1004 until all sensors have been sampled. The paired STM32G03 then combines the data from its four sensors to create a complete frame of data, which is sent to the MCU section. Each FDC1004 is given 50 ms to complete its sampling and data transfer.

#### 2.3.4. Noise Mitigation

The FDC1004 capacitance-to-digital converters have a shield trace generator, which is used to wrap the Sensor Board’s sensing pads in an active shield to protect against environmental interference and parasitic capacitance. This shield trace is kept at the same voltage as the excitation signal going to the sensing pads, preventing current leakage, and maintaining sensitivity and longer sensing range. However, some parasitic capacitance is generated between the bottom of the pad and the ground, which is addressed through background subtraction. To further minimize interference, thin PCB traces and interweaved routing are used to connect the FDC1004s to the Samtec connectors. The thin traces reduce stray capacitance and Electromagnetic Interference (EMI), while the interweaved routing maximizes isolation between the FDC1004s’ individual sensors.

#### 2.3.5. Calibration

The data collected from the Sensor Board’s individual sensing pads vary due to their varying distances between each FDC1004 capacitance-to-digital converter and the sensing pad. This issue was addressed by using hardware-programmed offsets, as suggested in the FDC1004′s datasheet [25], through a series of I2C commands. Each sensor was calibrated until its reading reached 1.0 pF. This hardware-based calibration is supplemented by software-based background estimation and subtraction. This involves measuring the “empty state” or background capacitance without a hand present over the sensor pads. After powering up the system, 50 consecutive measurements are collected and averaged to construct the background estimate, which is then subtracted from all subsequent data collected. During real-world use, the background estimation may drift due to various factors. This can be mitigated through periodic recalibration, which involves taking new baseline readings when it is known that no hand is present. Another potential method is to compute a rolling average of all capacitance readings since powering up and use this long-term average as the baseline. This is likely to be more effective as the time when a hand is present is likely to be much shorter than the empty state.

### 2.4. Classifier

The Classifier receives data from the Motherboard and predicts what gesture is being made. The input data is a 1×104 vector of capacitive values. The Classifier transforms the input data into a 1×5 vector of gesture class-membership values. The class corresponding to the largest value is chosen as the predicted gesture. In our work, Decision Tree, Naïve Bayes, Multi-layer Perceptron (MLP) neural network, and Convolutional Neural Network (CNN) classifiers were trained, and their performance was evaluated.

#### 2.4.1. Decision Trees

A Decision Tree classifier is a non-parametric classifier, which consists of a root node, internal nodes, and leaf nodes [27]. We decided to evaluate a Decision Tree classifier because it can perform multi-class classification, is easy to interpret, and is relatively fast to train. We used the Classification and Regression Tree (CART) algorithm to implement our Decision Tree classifier. The algorithm used can be summarized as follows: (1) calculate the weighted average of the Gini impurity for each sensing pad’s value; (2) use the smallest calculated value as the node; and (3) repeat for all remaining sensing pads’ values until all sensing pads have been evaluated.

#### 2.4.2. Naïve Bayes

A Naïve Bayes classifier is a probabilistic-based classifier, which uses Bayes’ theorem while assuming that the features in the input data are all independent (a strong, or “naïve” assumption) [28]. We decided to evaluate a Naïve Bayes classifier because it can perform multi-class classification, is easy to implement, and is fast to train. We used a Gaussian Probability Density Function (PDF) to implement our Naïve Bayes classifier. The algorithm used can be summarized as (1) calculate each gesture’s proportions, sample mean, and sample standard deviation; and (2) substitute the calculated values into the Naïve Bayes formula.

#### 2.4.3. Multi-Layer Perceptron Neural Network

**Figure 5:**
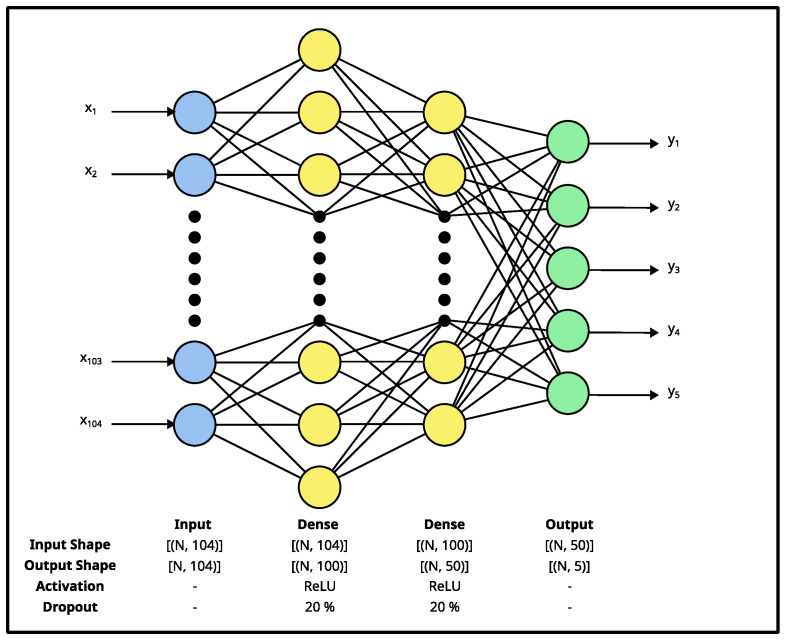
MLP classifier’s architecture. The network consists of six layers: an input dense layer, two dense + dropout blocks, and a dense output layer. The input layer has 104 neurons, the first hidden layer has 100 neurons, the second layer has 50 neurons, and the output layer has 5 neurons. A dropout rate of 20% was used for each dropout layer. The network takes a 1×104 vector (x_1_–x_104_) and produces a 1×5 vector (y_1_–y_5_). The network’s architecture was experimentally determined using a grid-search-based approach.

A Multi-Layer Perceptron (MLP) classifier is a neural-network-based classifier, which consists of densely connected layers that process 1D input data [29]. We decided to evaluate an MLP classifier because it can perform multi-class classification, is relatively easy to train, and can model complex, non-linear relationships. Figure 5 illustrates the MLP used in our work. The network’s architecture was experimentally determined using a grid-search-based approach; where, the number of layers, each layer’s type, and each layer’s attribute were increased/changed until the validation accuracy did not improve during training by further changes.

The MLP was trained for 100 epochs with a batch size of 16. A Sparse Categorical Cross-Entropy loss function was used to compute the loss. A Stochastic Gradient Descent (SGD) with a momentum optimizer was used to update the network’s weights. An initial learning rate of 0.001 and a momentum value of 0.9 were used.

#### 2.4.4. Convolutional Neural Network

**Figure 6:**
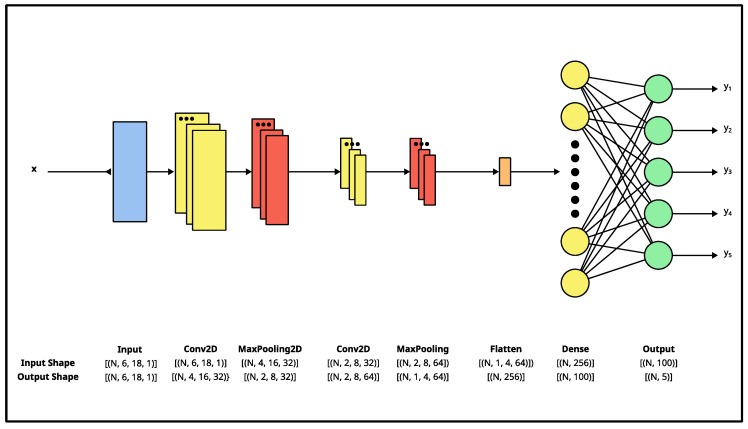
CNN classifier’s architecture. The network has eight layers: an input convolutional layer, two convolutional + pooling blocks, and an output dense layer. Each layer’s input and output are specified using the “NHWC” format: (batch size, height, width, channels), where appropriate. The network takes in a 6×18×1 matrix (**x**) and produces a 1×5 vector (y_1_–y_5_). The network’s architecture was experimentally determined using a grid-search-based approach.

A Convolutional Neural Network (CNN) is a neural-network-based classifier, which consists of convolutional layers that process 2D input data [30]. We decided to evaluate a CNN classifier because it can perform multi-class classification and is suited to data with a grid-like structure. Figure 6 illustrates the CNN used in our work. The network’s architecture was experimentally determined using a grid-search-based approach; where, the number of layers, each layer’s type, and each layer’s attribute were increased/changed until the validation accuracy did not improve during training by further changes.

The CNN was trained for 100 epochs with a batch size of 16. A Sparse Categorical Cross-Entropy loss function was used to compute the loss. A Stochastic Gradient Descent (SGD) with a momentum optimizer was used to update the network’s weights. An initial learning rate of 0.001 and a momentum value of 0.9 were used.

### 2.5. Data Acquisition

**Figure 7:**
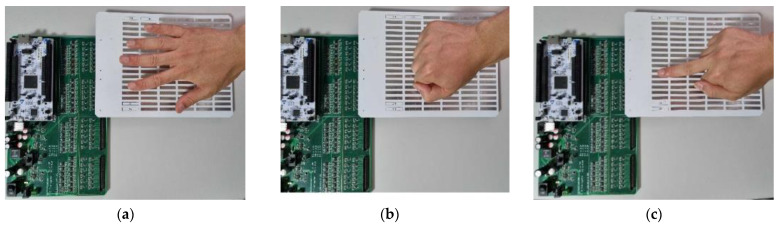

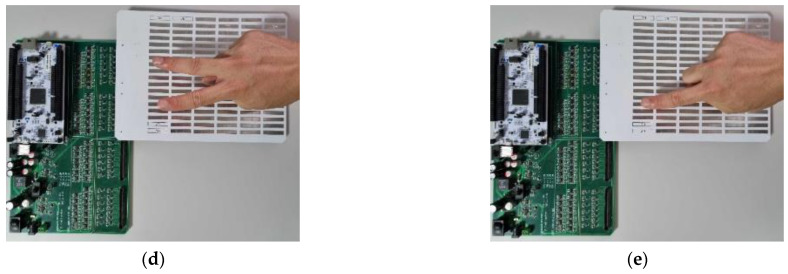
The five hand gestures used in our work: (**a**) Palm, (**b**) Fist, (**c**) Middle, (**d**) OK, and (**e**) Index.

The calibrated system was used to measure the capacitance of five test participants as they performed five different hand gestures (see Figure 7) 200 times. This resulted in a total of 5000 samples being collected. The variable of interest was the difference in the size and shape of the participants’ hands. To ensure the integrity of the data, the experiments were conducted on separate days for each participant in a controlled environment.

The gestures used combine a mixture of similar gestures (Middle, OK, and Index) and non-similar gestures (Palm and Fist) to thoroughly evaluate each classifier’s performance. For example, one would expect a classifier to have more difficulty with the Middle, OK, and Index gestures than with the Palm and Fist gestures. The collected data were subsequently used to train, validate, and test various classifiers to identify the hand gestures.

### 2.6. Training

To train the classifiers used in our work, we used a K-fold-like approach. The collected samples were divided into Participants 1, 2, 3, 4, and 5 sets. Each classifier was trained five times, each time using a different Participant set as the test data and the remaining sets as the training and validation sets. The training and validation sets were randomly shuffled and divided up into a ratio of 80:20, where 80% of the data was used for training and 20% was used for validation. Table 2 tabulates the combination of participant training and validation sets, and the test set used.

### 2.7. Evaluation

To evaluate each model’s performance, accuracy, precision, recall, and the balanced F-score ($F_{1}$ score) were used.

Accuracy is defined as:

(2) $$A=\frac{TP+TN}{TP+FP+TN+FN}\times 100\;\%,$$

where TP denotes true positives, TN denotes true negatives, FP denotes false positives, and FN denotes false negatives.

Precision measures the proportion of positives that were correct. It is defined as:

(3) $$P=\frac{TP}{TP+FP}\times 100\;\%,$$

where TP denotes true positives, and FP denotes false positives.

Recall measures the proportion of actual positives that were identified correctly. It is defined as:

(4) $$R=\frac{TP}{TP+FN}\times 100\;\%,$$

where TP denotes true positives, and FN denotes false negatives. The $F_{1}$ score is the harmonic average of Precision and Recall. It is defined as:

(5) $$F_{1}=2\cdot \frac{P\cdot R}{P+R}\times 100\;\%,$$

where P denotes precision, and R denotes recall. To compute each model’s final precision, recall, and $F_{1}$ score, each class’s values were computed, and then averaged.

As discussed, each classifier was trained five times, each time using a different Participant set as the test data and the remaining sets as the training and validation sets (please see Table 2). At each iteration the classifier was tested; afterwards, a confusion matrix was prepared and the accuracy, precision, recall, and $F_{1}$ score computed. Here, the sum of the confusion matrices is referred to as the “combined confusion matrix”; the average of the computed values is referred to as the “average” value.

## 3. Results

In this section, the results from testing the trained Decision Tree, Naïve Bayes, MLP neural network, and CNN classifiers are presented and described.

### 3.1. Decision Tree

**Figure 8:**
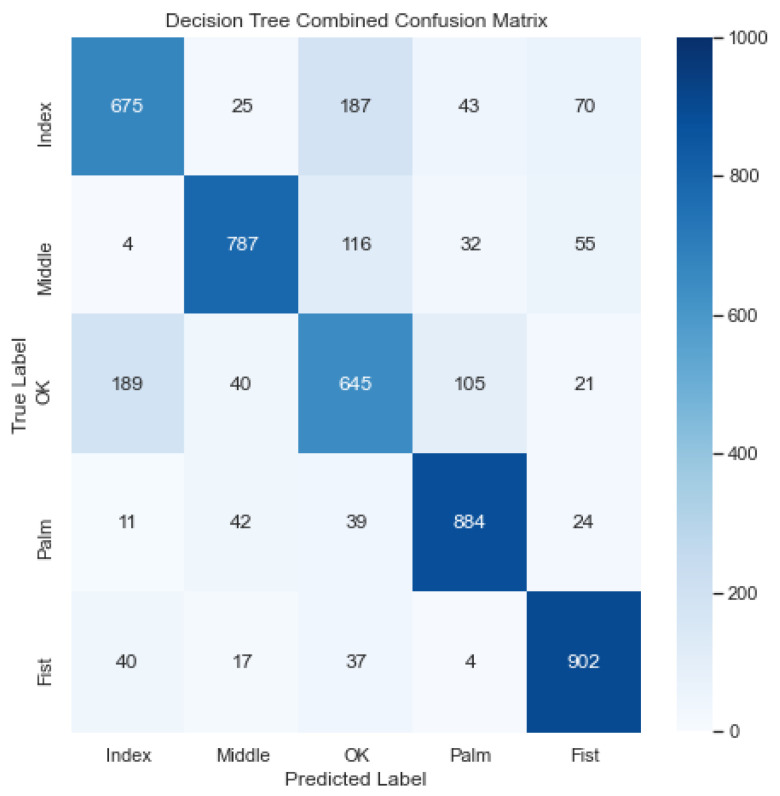
Combined confusion matrix for decision tree model. Rows represent actual classes; columns represent predicted classes. Dark blue shading indicates a large value; light blue shading indicates a small value. The average accuracy, precision, recall, and $F_{1}$ score for this model were 91.18%, 77.95%, 77.96%, and 77.85%, respectively.

Figure 8 illustrates the combined confusion matrix for the Decision Tree model. The average accuracy, precision, recall, and $F_{1}$ score for this model were 91.18%, 77.95%, 77.96%, and 77.85%, respectively. Considering Figure 8, it looks like the model classified the gestures adequately, but confused the OK gesture with the Index and Middle gesture a lot.

### 3.2. Naïve Bayes

**Figure 9:**
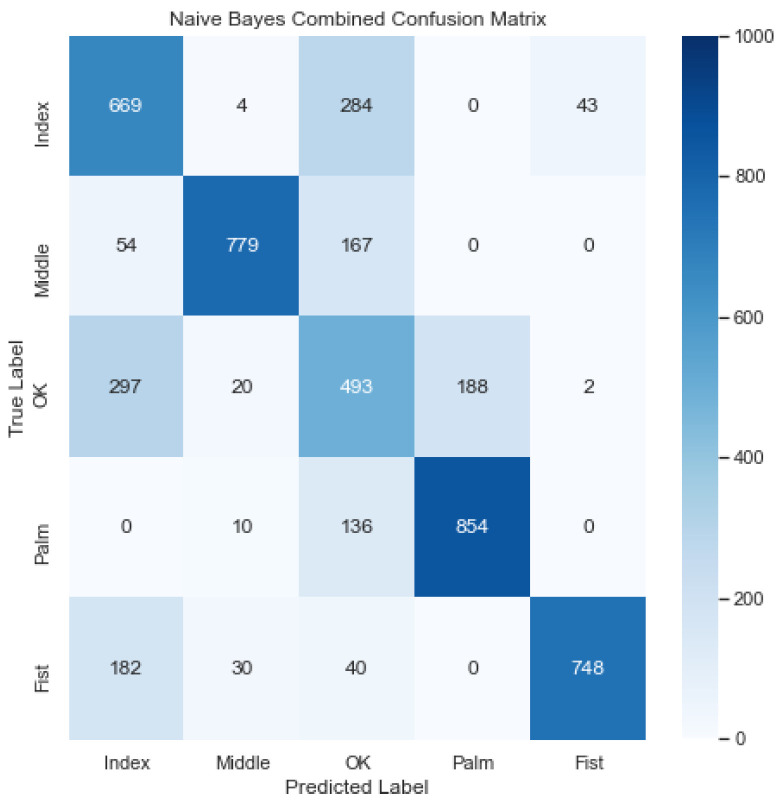
Combined confusion matrix for Naive Bayes model. Rows represent actual classes; columns represent predicted classes. Dark blue shading indicates a large value; light blue shading indicates a small value. The average accuracy, precision, recall, and $F_{1}$ score for this model were 88.34%, 73.68%, 70.86%, and 71.78%, respectively.

Figure 9 illustrates the combined confusion matrix for the Naïve Bayes model. The average accuracy, precision, recall, and $F_{1}$ score for this model were 88.34%, 73.68%, 70.86%, and 71.78%, respectively. Considering Figure 9, it looks like the model classified the gestures poorly, confusing the OK gesture with the Index and Middle gesture a lot.

### 3.3. Multi-Layer Perceptron (MLP)

**Figure 10:**
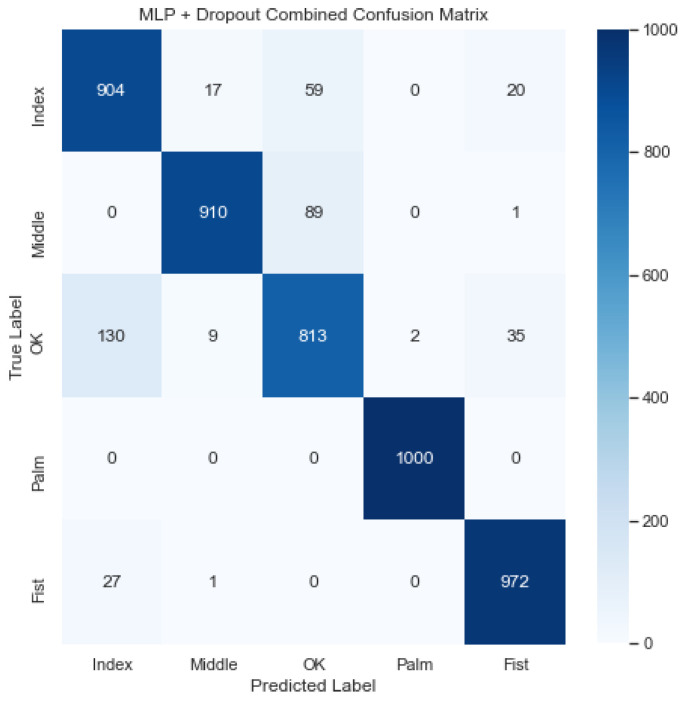
Combined confusion matrix for MLP model. Rows represent actual classes; columns represent predicted classes. Dark blue shading indicates a large value; light blue shading indicates a small value. The average accuracy, precision, recall, and $F_{1}$ score for this model were 96.87%, 92.25%, 92.16%, and 92.16%, respectively.

Figure 10 illustrates the combined confusion matrix for the MLP model. The average accuracy, precision, recall, and $F_{1}$ score for this model were 96.87%, 92.25%, 92.16%, and 92.16%, respectively. Considering Figure 10, it, again, looks like the model classified the Palm and Fist gestures accurately, but struggled to classify the OK gesture, confusing it, again, with the Index and Middle gestures.

### 3.4. Convolution Neural Network (CNN)

**Figure 11:**
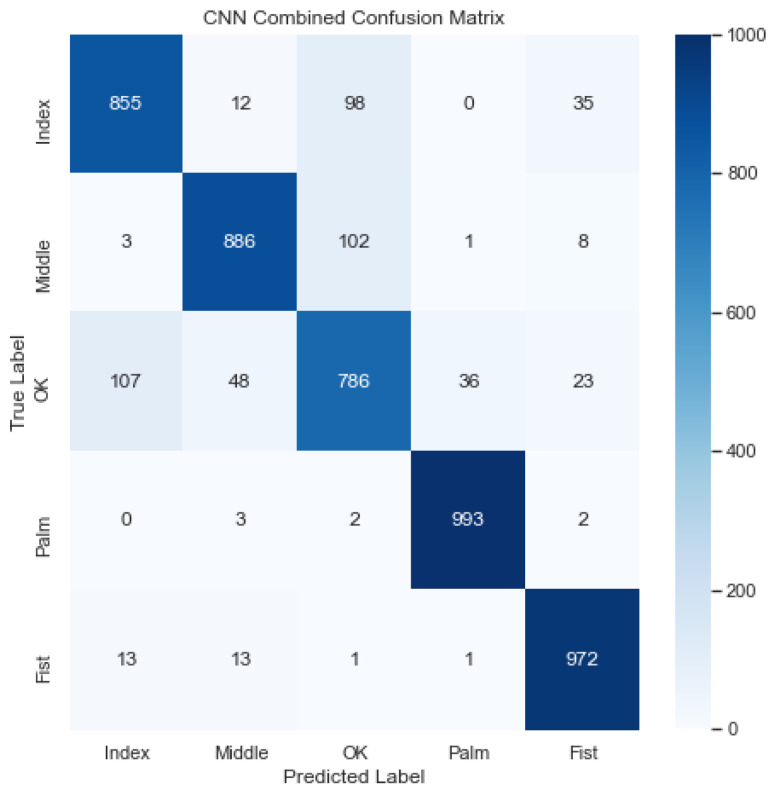
Combined confusion matrix for CNN model. Rows represent actual classes; columns represent predicted classes. Dark blue shading indicates a large value; light blue shading indicates a small value. The average accuracy, precision, recall, and $F_{1}$ score for this model were 95.94%, 89.75%, 89.84%, and 89.77%, respectively.

Figure 11 illustrates the combined confusion matrix for the CNN model. The average accuracy, precision, recall, and $F_{1}$ score for this model were 95.94%, 89.75%, 89.84%, and 89.77%, respectively. Considering Figure 11, it looks like the model classified the Palm and Fist gestures accurately, but struggled to classify the OK gesture, confusing it with the Index and Middle gestures.

### 3.5. Performance Comparison

Table 3 tabulates the average metrics achieved by each model. The MLP model performed the best, achieving an average accuracy, precision, recall, and $F_{1}$ score of 96.87%, 92.25%, 92.16%, and 92.16%, respectively. The Naïve Bayes model performed the worst, achieving an average accuracy, precision, recall, and $F_{1}$ score of 91.18%, 73.68%, 70.86%, and 71.8%, respectively. The neural-network-based models (MLP and CNN) all performed better than the other models (Decision Tree and Naïve Bayes). From the combined confusion matrices, it is evident that the models classified the Palm and Fist gestures more consistently than the Index, Middle, and OK gestures.

As can be seen from Table 1, our proposed system is more accurate than the recently reported hand gesture recognition systems. While some of those works can recognize more gestures, most of those systems are not able to identify static hand gestures. The novel work reported by Wei et al. [18] is based on capacitive sensing and like our proposed system, can recognize static hand gestures. Its operating Sensor–Hand distance (6–9 cm) is like ours (5–8 cm). However, their system was trained and evaluated on the same group of (four) subjects and not with “unseen” or unregistered subjects, the subjects who were not part of the training dataset. The classification accuracy for a 50–50 train–test split is 91.6%. If we train and test our proposed system in a similar manner, our system’s accuracy is 100%. However, during real-world operation, a gesture recognition system needs to identify the gestures of unregistered subjects. Therefore, our proposed system was tested with “unseen” subjects and achieved an accuracy of 96.87%. It should be noted that Wei et al. trained their capacitive system to recognize four static gestures, whereas our proposed system was trained to identify five gestures. One of the strong features of the work of Wei et al. is their system’s ability to recognize dynamic hand gestures, which is a current limitation of our system.

## 4. Conclusion and Future Works

In conclusion, we successfully developed a capacitive sensing and neural-network-based hand gesture recognition system, which recognized gestures with a best average accuracy of 96.87%.

The proposed system was used to capture five gestures of five participants to create a dataset of 2D gesture “capacitive images”. The dataset was then used to train Decision Tree, Naïve Bayes, Multi-Layer Perceptron (MLP) neural network, and Convolutional Neural Network (CNN) classifiers. Each classifier was trained five times; each time, the classifier was trained using four different participants’ gestures and tested with one different participant’s gestures.

After training, the Naïve Bayes and Decision Tree classifiers achieved average $F_{1}$ scores of 71.78% and 77.85%, respectively, and the MLP and CNN classifiers achieved average $F_{1}$ scores of 92.16% and 89.77%, respectively. Our results show that the neural-network-based classifiers outperformed the other classifiers, and that the MLP classifier outperformed all the other classifiers.

It is interesting that the MLP classifier outperformed the CNN classifier. In general, a CNN is expected to outperform an MLP. This is because CNNs are designed to process spatial data, e.g., images, by exploiting the data’s structure. We reason that due to the 6×18 resolution of the data captured, the CNNs pooling layers may have reduced the data’s dimensionality too much, removing important features.

We observed that all classifiers were able to differentiate between non-similar gestures (Palm and Fist) relatively easily; however, they were less able to differentiate between similar gestures (Middle, OK, and Index), with OK having the largest confusion observed, on average. We reason this was due to the Sensor Board’s sensing pads’ physical size and layout, limiting the spatial resolution of the collected data, which, in turn, made it harder for the classifiers to differentiate between similar gestures.

In the future, we plan to capture gestures from more users, use more gestures, use more non-similar gestures, capture left- and right-hand gestures, and investigate the amended dataset’s effects on the system’s performance; capture dynamic gestures and investigate the system’s performance when using a Recurrent Neural Network (RNN) to classify gestures; use alternative capacitive sensing modes, e.g., shunt mode, and investigate the system’s performance; and implement a Field-Programmable Gate Array (FPGA)-based design to optimize our system’s performance.

## Figures and Tables

**Table 1:** The proposed system benchmarked against a collection of recent works on hand gesture recognition. It is one of the few systems that is capable of accurately identifying static hand gestures.

Article	Sensing Method	Participant Details	No. of Gestures (Type)	Accuracy (%)	Sensor–Hand Distance (cm)	Remarks
Duan et al. [10] (2020)	Ambient light sensing using PD	Trained with 4 subjects, tested on 3 “unseen”	7 (Dynamic)	82.57–85.67	10	Accuracy is 96.83% if trained and tested (90–10 split of data) on the same set of 4 subjects.
Yu et al. [4] (2021)	IR	Trained with 4 and tested on 1 “unseen”	8 (Dynamic)	92.13	20	Significantly degraded accuracy with Visible light.
Ma et al. [11] (2022)	Solar cell	Trained with 2 subjects, tested on 1 “unseen”	6 (Dynamic)	94	10	99% accuracy if trained and tested on the same subject.
Skaria et al. [16] (2019)	Micro-Doppler Signatures	Trained with 1 subject, tested on 1 “unseen”	14 (Dynamic)	45.50–51.20	10–30	When trained and tested on the same subject, accuracy ranged between 90.30% and 95.50%.
Tian et al. [13] (2018)	Wireless (Wi-Fi)	10	9 (Dynamic)	~	50–250	Accuracy is 96% when trained and tested (train–test split not given) on the same set of 10 subjects. Can recognize two-handed gestures
Singh et al. [9] (2016)	Capacitive sensing	5	16 (Dynamic)	~	<10	Accuracy is 93% when trained and tested (train–test split not given) on the same set of 5 subjects.
Wei et al. [18] (2019)	Capacitive sensing	4	4 (Static)	~	6–9	Accuracy is 91.6% when trained and tested (50–50 split of data) on the same set of 4 subjects.
Noble et al. Proposed (2022)	Capacitive sensing	Trained with 4 and tested on 1 “unseen”	5 (Static)	96.87	5–8	Accuracy is 100% if trained and tested (90–10 split of data) on the same set of 5 subjects.

**Table 2:** Training, validation, and test sets. Each classifier was trained five times, each time using a different Participant set as the test data and the remaining sets as the training and validation sets.

Iteration	Participant Training and Validation Sets	Participant Testing Set
1	2, 3, 4, 5	1
2	1, 3, 4, 5	2
3	1, 2, 4, 5	3
4	1, 2, 3, 5	4
5	1, 2, 3, 4	5

**Table 3:** Performance comparison among the classifier models used for the proposed hand gesture recognition system. The MLP classifier is the best performing across all performance metrics.

Model	Average Accuracy	Average Precision	Average Recall	Average *F*_1_ Score
Decision Tree	91.18%	77.95%	77.96%	77.85%
Naïve Bayes	88.34%	73.68%	70.86%	71.78%
MLP	**96.87%**	**92.25%**	**92.16%**	**92.16%**
CNN	95.94%	89.75%	89.84%	89.77%

## Data Availability

The data presented in this study are available on request from the corresponding author.
